# Association between maternal Autism Spectrum Quotient scores and the tendency to see pragmatic impairments as a problem

**DOI:** 10.1371/journal.pone.0209412

**Published:** 2018-12-19

**Authors:** Kaori Hanabusa, Manabu Oi, Naotake Tsukidate, Yuko Yoshimura

**Affiliations:** 1 Department of Child Development, United Graduate School of Child Development, Kanazawa University, Kanazawa, Ishikawa, Japan; 2 Research Center for Child Mental Development, Kanazawa University, Kanazawa, Ishikawa, Japan; 3 Department of Humanities, Yamanashi Eiwa College, Kofu, Yamanashi, Japan; 4 Faculty of Education, Institute of Human and Social Sciences, Kanazawa University, Kanazawa, Ishikawa, Japan; Newcastle University Institute for Health and Society, UNITED KINGDOM

## Abstract

The aim of the present study was to test the hypothesis that individuals with higher Autism Spectrum Quotient (AQ) scores would be more permissive of pragmatic impairments than those with lower AQ scores. We investigated the presence of a correlation between the AQ scores of mothers with children in grades 1 to 6 and their evaluation of assumed pragmatic impairments in children using the Maternal Evaluation of Pragmatic Impairments in Children (MEPC) measure. Mothers were asked to rate how they would feel if their child showed the communication behaviors listed in scales D (coherence), E (inappropriate initiation), F (stereotyped language), G (use of context), and H (nonverbal communication) of the Children’s Communication Checklist-2, which measures pragmatic impairments. All responses were given on a five-point Likert scale. The results indicated that the higher the maternal AQ score, the less the mother tended to evaluate pragmatic impairments as a problem. We also examined whether the age and gender of assumed children influenced the correlation between AQ and MEPC scores, but found no significant correlation. The partial correlation coefficients were calculated for each subscale, none of which was significant. A negative correlation was found between AQ and MEPC scores as a whole.

## Introduction

Pragmatics relates to the ability to use language for communication. It is generally agreed that difficulties in the domain of pragmatics are a universal feature of autism [1]. It is widely reported that even the most verbally capable autistic individuals fail to understand non-literal speech such as sarcasm, joking, and metaphorical expressions [2]. Furthermore, Oi’s [3] review of research on pragmatic impairments in individuals with high-functioning pervasive developmental disorders noted that a wide variety of pragmatic impairments, including speech acts, expression of mental states, comprehension of indirect speech, questions and answers, conversational turn-taking, narratives, pronoun and address forms, inferential language, and reference and cohesion, has been examined. Perkins [4] defines pragmatic impairment as emergent or compensatory adaptations of difficulties such as cognition, language, and/or sensory movement.

Consequently, because of innate differences between individuals with autism spectrum disorder (ASD) and typically developing (TD) people, communications between them do not generally go smoothly in social settings. It seems that individuals with ASD and TD individuals cannot empathize with one another because of the pragmatic impairments of individuals with ASD.

Oi [5] reported that in social pragmatic groups, adult TD leaders who were unfamiliar with ASD were unable to ensure opportunities for children with high-functioning ASD to learn social skills because they had been perplexed at and reacted too sensitively to their pragmatic impairments. Abele and Grenier [6] noted that it is difficult for TD people to be matter-of-fact about social conventions toward individuals with pragmatic impairments before becoming irritated.

Individuals with pragmatic impairments are not well understood by TD people, and have therefore been on the receiving end of substantial impatience, scolding, and irritation [6]. The relationship with behavioral problems is much stronger for pragmatic language impairment than for specific language impairment [7,8]. Pragmatic language impairment such as difficulties initiating a conversation and taking turns and developing a topic while having a conversation can restrict children from communicating their thoughts and needs [8,9]. Rodas et al. [9] examined how structural and pragmatic language predicted later child anxiety and externalizing behaviors. They found that children with a discrepancy between structural and pragmatic language skills may be most at risk of experiencing anxiety. Therefore, what type of condition enables individuals with pragmatic impairments to execute smooth communication?

Clark [10] stated that language use, i.e., pragmatics, is a form of joint action. Although speaking and listening are autonomous processes in unilateral accounts, speaking and listening together form a joint activity in bilateral accounts [11]. Perkins [4] mentioned that pragmatics is inherently interpersonal, in other words, the outcome of joint engagement between two or more interactants that cannot be entirely reduced to the distinct capacities of individuals.

Ayaya [12] mentioned that communication between people diagnosed with communication disorders could be established without any problems. She was diagnosed as ASD after adulthood and described her experience when she joined a self-help group as follows. Although people diagnosed as having communication disorders had gathered, communication was established without any problems, and they were excited to talk in a group about the experience of feeling alienated. In addition, she mentioned that listening to the difficulties of others enabled her to comprehend her own difficulties.

Komeda et al. [13] found that people with autism empathize with autistic characters. They conducted judgement tasks capable of indexing self-relevant processing in individuals with and without ASD. The results demonstrated that the ventromedial prefrontal cortex (vmPFC) was significantly activated in individuals with ASD in response to autistic characters and in TD individuals in response to non-autistic characters. They suggested that both individuals with ASD and TD individuals make selective neural responses toward similar others.

In the present study, we focused on the following points regarding how individuals with ASD reduce pragmatic impairments. The first point is empathy among similar people. A hypothesis that explains an individual’s being permissive to or empathizing with the characteristics of another that are similar to their own is called the “similarity hypothesis”. The similarity hypothesis states that perceivers empathize with targets similar to themselves, which consequently facilitates cognitive processing [14]. Komeda [14] focused on similarities between a perceiver’s personality traits and ASD-related characteristics and concluded that people with ASD empathize with others with ASD according in accordance with this hypothesis. In the case of communication between people with ASD, pragmatic impairments may not be problematic. Another point is based on our clinical experience that people with ASD cannot recognize the state of their communication partner. From the standpoint of TD individuals, certain specific conditions are regarded as pragmatic impairments, but this may not be a problem when ASD people communicate with each other. That leaves the question, does the same thing happen between individuals with the broad autism phenotype (BAP) and those with ASD?

The BAP is associated with a group of ‘sub-threshold’ social skills and communication traits and unusual personality features that are frequently found in the relatives of people with autism, and that are believed to be milder manifestations of traits characteristic of clinically diagnosed autism [15]. Some research findings suggest that parents and siblings of autistic probands have significantly greater difficulty using language to communicate for social purposes (pragmatics) compared with controls [15]. Bishop et al. [16] examined the BAP in parents of children with ASD, and found that while parents of probands with ASD obtained relatively high scores (indicative of a more autistic-like profile) on the communication subscale of the Autism Spectrum Quotient (AQ) [17], which was developed by Baron-Cohen and colleagues as a brief, self-administered instrument for assessing the BAP in individuals with a normal intelligence quotient (IQ) [17], they did not differ from other parents in language abilities, as assessed by the short-form verbal IQ test. They concluded that this finding suggests a dissociation between the ability to learn words and their meanings and the ability to use language skills to communicate and socialize effectively. Oi et al. [18] conducted a large national survey for the Japanese version of the Children’s Communication Checklist-2 (CCC-2) [19] and reported that aspects of communicative impairment measured by the CCC-2 are continuously distributed, and that ASD and language impairment fit inside the bell curve of the General Communication Composite of the CCC-2; these findings suggest, at least in part, that individuals with the BAP have pragmatic impairments.

We sought to investigate the correlation between the degree to which people generally view pragmatic impairments as a problem and the degree of that person’s own BAP. To accomplish this, we decided to use AQ scores to measure the degree of BAP in general mothers, and an item from the domain related to pragmatic impairments in the Japanese version of the CCC-2 [20] for the evaluation. The reason we decided to target children was because no examination had been standardized for measuring pragmatic aspects among adults in Japan at the time of implementation; however, the Japanese version of the CCC-2 had already been standardized by Tsukidate et al. [20]. The evaluation for pragmatic impairments in children who were assumed to have pragmatic impairments, not for children with actual pragmatic impairments, was made on a five-point Likert scale, from 1) “It is not a big problem” to 5) “It is a big problem”. In the Japanese version of the CCC-2, difficulties in communication were found to decrease with age. At the same time, the higher a child’s age, the greater the pragmatic impairments tended to be estimated as being problematic. Furthermore, girls had lower raw scores than boys in all subscales; i.e., their language and pragmatic skills were high. Tsukidate et al. [20] suggested that the differences between men and women were not discussed in the British and translated versions from other countries, but may be one of the features of the Japanese version. In assumed children, there was a possibility that boys would be evaluated more severely than girls. Also, in assumed children, it was possible that higher-grade students would be evaluated more severely than lower-grade students. Therefore, we considered that it was necessary to investigate the difference between whether the assumed children were boys or girls, and whether they were higher- or lower-grade students.

The objectives of the present study were to investigate whether there is a correlation between the mother’s AQ score and their evaluation of an assumed child’s pragmatic impairments, and to confirm the presence or absence of a secondary influence on the possible correlation between the age and gender difference of assumed children.

Thus, we formulated the following three hypotheses. First, mothers with higher AQ scores would be more permissive of assumed pragmatic impairments in their child. Second, mothers would more frequently tend to evaluate assumed pragmatic impairments as a problem as their child got older. Third, mothers would more frequently tend to evaluate assumed pragmatic impairments as a problem when their children were boys.

## Methods

### Participants

In the present study, we selected general mothers who had a child of the same age as that of CCC-2 for participants to rate the assumed pragmatic impairments of a child. Although the presumed evaluators of the CCC-2 were parents (and adults close to parents, such as teachers and child caregivers), to control the condition, we chose mothers, who have a high proportional responsibility for childcare in Japan. The study participants were 100 mothers randomly selected by a marketing research corporation in Japan according to sample allocation from over 800,000 questionnaire respondents whose ages were from in the teens to over 60 years at the time of data collection. We applied sample allocation to choose eight or nine mothers of children of each gender in each grade from grades 1 to 6.

The mothers ranged in age from 26–55 years, with a mean of 40.3 years (standard deviation [SD] = 5.3). The mothers’ most recently completed educational backgrounds were classified as follows: 0 as junior high school, 29 as high school, 29 as junior college, and 42 as university. According to the results of the 2010 national census, the mean proportions regarding the latest educational backgrounds of married Japanese women aged 25–54 years (*n* = 24,740,251) were 3.9% junior high school, 33.5% high school, 27.7% junior college, and 16.8% university [21]. A chi-square test was conducted to compare participants in the present study with figures from the national census; as a result, a significantly higher proportion of individuals in the present study had a university education (*p* < 0.01).

Regarding the employment status of mothers, 51 were full-time housewives, 28 were part-time workers, 15 were full-time workers, and six were engaged in an independent or family business. We then referred to the longitudinal survey of newborns in the twenty-first century (2001 Cohort) conducted by the Japanese Ministry of Health, Labour and Welfare (*n* = 27,101), which was conducted continuously every year from 2001 (first) to 2015 (15th). According to the seventh survey, when their child was a first grader (6 years old), 42.8% of the mothers were full-time housewives, 30.2% were part-time workers, 17.3% were full-time workers, and 6.0% were engaged in an independent or family business [22]. In the 12th survey, when their child was a sixth grader (12 years old), 26.0% of the mothers were full-time housewives, 45.0% were par-time workers, 20.8% were full-time workers, and 7.9% were engaged in an independent or family business [23]. The proportion of “employed” mothers increased year-by-year from the seventh through the 12th surveys. We then calculated the average values from the seventh to the 12th surveys because our sample included first to sixth graders, and conducted a chi-square test to compare data from the present study with those of the longitudinal survey. Compared with the longitudinal national survey data, a significantly lower proportion of mothers in the present study were employed *(p* = 0.01). This result might reflect the fact that the participants were questionnaire respondents from an online survey conducted by a research company.

### Procedures

From the extracted mothers, responses were obtained in a questionnaire format. All of the questionnaires could be accessed online at home using a personal computer. The questionnaires started by collecting demographic information and explaining ethical approval, followed by asking the respondent to answer all subscales of the AQ, and finally, assuming that the pragmatic impairment symptoms on the CCC-2 subscales could be seen in their children, asking the mothers to rate on a five-point Likert scale to what extent they felt these pragmatic impairments were a problem. Questionnaires were presented in order, and if the participants did not agree with the ethical approval or provide appropriate answers, the following items were not presented and the questionnaire was considered finished. If a participant did not fully complete the questionnaire, the research company was to conduct the questionnaire on another respondent in order to maintain sample allocation and a population of 100 people.

This study was approved by the medical research ethics committee at Kanazawa University and performed in accordance with the ethical standards laid down in the 1964 Declaration of Helsinki and its later amendments.

### Measures

We asked mothers to rate how they would feel in general if their child showed the pragmatic impairments listed in the 25 items from the five subscales of the CCC–2 on a five-point Likert scale to measure permissiveness regarding assumed pragmatic impairments in their children. We measured maternal AQ at the same time to investigate the correlation between maternal AQ and permissiveness regarding assumed pragmatic impairments in their children.

#### Japanese version of AQ-revised (Wakabayashi, 2016)

Wakabayashi et al. [24] published the Japanese version of the AQ in 2004. Some of the Japanese wording was later revised [25]. In the present study, we utilized the 2016 version. The AQ consists of the following five subscales: social skills, attention switching, local detail, communication, and imagination. Each subscale contains 10 items, resulting in a total of 50.

#### Maternal Evaluation of Pragmatic Impairments in Children (MEPC)

The MEPC is an ad hoc creation from the CCC-2. The CCC-2 is one of the assessment tools used to evaluate children’s communicative ability, including pragmatic aspects. The CCC-2 consists of 10 subscales from A to J to identify what kinds of difficulties a child may have in terms of communication. In the MEPC measure, the mothers provide evaluations under the assumption that their child has pragmatic impairments. Mothers were asked to rate how they would feel if their child showed any of the pragmatic impairments listed in subscales D (coherence), E (inappropriate initiation), F (stereotyped language), G (use of context), and H (nonverbal communication) of the CCC-2, which measure pragmatic impairments, on a five-point Likert scale as follows: “1. It is not a big problem”; “2. It is only a small problem”; “3. Neutral”; “4. It is somewhat of a problem”; and “5. It is a big problem”. Each subscale contains five items, resulting in a total of 25.

We also examined similar items of the AQ and subscales D to H in the CCC-2 and determined that there were similarities between the following three pairs of items: no. 18 of the AQ (communication subscale), “When I talk, it isn’t always easy for others to get a word in edgeways” and no. 35 of the CCC-2 (E subscale), “It’s difficult to stop him/her from talking”; no. 35 of the AQ (communication subscale), “I am often the last to understand the point of a joke”, and no. 15 of the CCC-2 (G subscale),“Misses the point of jokes and puns (though may be amused by nonverbal humor such as slapstick)”; and no. 36 of the AQ (social skill subscale), “I find it easy to work out what someone is thinking or feeling just by looking at their face”, and no. 39 of the CCC-2 (H subscale), “Fails to recognize when other people are upset or angry”. These items were not excluded at the time of MEPC creation, but were subject to analysis.

### Data analysis

A chi-square test was conducted with the national survey on the educational level and employment status of the mothers to confirm the representativeness of the sample. One-way analysis of variance (ANOVA) was applied for both maternal AQ total and MEPC total scores, with the employment status of the mothers as the independent variable. We also applied a one-way ANOVA for both AQ total and the MEPC total scores, with gender and age differences in assumed children as independent variables.

Pearson’s correlational coefficients were calculated between maternal AQ total and the MEPC total scores. We used Pearson’s correlation coefficient because we assumed a linear relationship between MEPC and AQ scores. Furthermore, neither a ceiling nor a floor effect was observed for both MEPC and AQ scores, so the mean value was functioning properly as a representative value of the data. Each AQ subscale and MEPC total and subscale score was also analyzed in the same way. Cohen [26] provided guidelines for interpreting the magnitude of correlation coefficients. Small, moderate, and large effect sizes are operationalized as 0.10, 0.30, and 0.50, respectively [26]. The partial correlation between AQ total and MEPC total scores, and between the subscales of the AQ and MEPC, were calculated to check whether that the mother’s educational level influenced the correlation between AQ and MEPC scores. Subsequently, three pairs of similar items were excluded, and the correlations were calculated for the AQ total and MEPC total scores, the communication subscale of the AQ and MEPC total scores, and the imagination subscale of the AQ and MEPC total scores. Next, we conducted a Pearson’s correlation analysis to assess whether there was a correlation between AQ total and MEPC total scores for each gender and age of assumed children to confirm the presence or absence of a secondary influence on the possible correlation between the age and gender difference of assumed children. Finally, Cohen’s q, which compares independent sample correlations [27], was calculated to compare the correlation between AQ total and MEPC total scores for each gender and age of assumed children. Cohen’s q is reported as an index of the effect size. Values under 0.10 were categorized as having no effect size, 0.10–0.30 as small effect size, 0.31–0.50 as moderate, and ≥0.51 as large [26].

## Results

### Maternal AQ

The average maternal AQ total score was 18.82 (SD = 7.45). We compared the AQ total scores with the AQ subscale scores from the present study to those from women in several prior studies (Table 1).

**Table 1:** Mean scores on the AQ subscales among females in previous studies.

Study	Group	*n*	Mean age	Total AQ	Social skills	Attention switching	Local details	Communication	Imagination
Baron-Cohen, et al. [17]	Controls	98	37.0	15.4	2.3	3.6	5.4	2.1	1.9
	Control students	386	21.0	16.4	2.0	4.3	5.4	2.7	2.0
Bishop, et al. [16]	ASD mothers	65	40.0		2.22	3.55	3.89	2.12	2.20
	Control mothers	48	39.9		1.75	3.25	4.54	1.75	1.88
Wheelwright, et al. [28]	ASD mothers	1429	41.2	16.4	3.1	4.0	4.4	2.6	2.4
	Control mothers	658		13.1	1.9	3.1	4.7	1.7	1.8
Ruta, et al. [29]	ASD mothers	130	39.7	16.39	2.2	3.74	4.55	2.12	3.78
	Control mothers	150	38.9	14.51	1.8	3.69	4.49	1.69	2.84
Wakabayashi, et al. [24] *AQ Japanese ver*.	Company employees	91	33.6	17.9	3.1	4.3	5.0	2.6	2.9
	Students	495	20.3	19.9	3.7	5.1	4.8	3.5	2.8
Present study	Mothers of elementary school students	100	40.3	18.82	4.81	3.93	4.24	2.57	3.22

The scores obtained for the present sample were in good agreement with those of Wakabayashi et al. [24], who found that 3% of the subjects in a normal group scored more than 33 points (compared with 2% of the subjects in the present study); this could be considered a clinical cutoff score for ASD.

To check whether the AQ was affected by mother’s employment status, a one-way ANOVA with three levels of employment status for mothers (full-time housewives vs. part-time workers vs. full-time workers engaged in an independent or family business) was conducted with the AQ total scores to assess whether the mother’s employment status influenced the AQ. As a result, the main effect of employment status was not significant (F(2,97) = 1.13, *p* = 0.33). Therefore, there was no difference in the AQ total score depending on the mother’s employment status.

Table 2 shows the average AQ total scores according to the gender and age of assumed children.

**Table 2:** Average AQ total scores.

Gender of assumed children		Age of assumed children	
Boys (*n* = 50)	Girls (*n* = 50)	7–9 years (*n* = 52)	10–12 years (*n* = 48)
18.14	19.5	18.35	19.33

To check whether AQ scores were affected by the gender and age of the assumed children, first, a one-way ANOVA with two levels of the assumed children’s gender (boys vs. girls) was performed on AQ total scores. The main effect due to the gender of assumed children was not significant (F(1,98) = 0.82, *p* = 0.37). In addition, no difference was found in total AQ scores depending on the gender of assumed children. Another one-way ANOVA with two levels of the assumed children’s grade (7–9 years vs. 10–12 years) was performed on AQ total scores. The main effect due to the age of assumed children was not significant (F(1,98) = 0.80, *p* = 0.37). Therefore, there was no difference in total AQ scores depending on the age of assumed children.

### MEPC

The average maternal MEPC total score was 80.77 (SD = 19.99). To check whether MEPC scores were affected by mother’s employment status, a one-way ANOVA with three levels of mother’s employment status (full-time housewives vs. part-time workers vs. full-time workers engaged in an independent or family business) was conducted with the MEPC total scores to assess whether mother’s employment status influenced MEPC scores. As a result, the main effect due to mother’s employment status was not significant (F(2,97) = 0.49, *p* = 0.61). Therefore, there was no difference in MEPC total scores depending on the mother’s employment status.

Table 3 shows the average MEPC total scores according to the gender and age of assumed children.

**Table 3:** Average MEPC total scores.

Gender of assumed children		Age of assumed children	
Boys (*n* = 50)	Girls (*n* = 50)	7–9 years (*n* = 52)	10–12 years (*n* = 48)
84.08	77.46	77.7	84.1

To assess whether MEPC scores were affected by the gender and age of assumed children, a one-way ANOVA with two levels of assumed children’s gender (boys vs. girls) was performed on the MEPC total scores. The main effect due to the gender of assumed children was not significant (F(1,98) = 2.76, *p* = 0.10). Therefore, there was no difference in MEPC total scores depending on the gender of assumed children. In addition, another one-way ANOVA with two levels of assumed children’s grade (7–9 years vs. 10–12 years) was performed on the MEPC total scores. The main effect due to the age of assumed children was significant (F(1,98) = 8.04, *p* = 0.01). Therefore, there was a difference in MEPC total scores depending on the age of assumed children.

### Maternal AQ and MEPC scores

Based on Pearson’s correlation coefficients, a significant negative correlation was found between maternal AQ total and MEPC total scores (*r* = –0.26, *p* < 0.01) (S1 Fig). The partial correlation coefficient for the AQ total and MEPC total scores, excluding the influence of the mother’s educational background, was calculated, but the significant difference (*r* = –0.26, *p* < 0.01) remained. There was almost no change in the partial and original correlation coefficients. When maternal AQ scores were higher, mothers evaluated pragmatic impairments as less of a problem.

Table 4 shows the correlation coefficients and 95% confidence intervals between the AQ subscale scores and the MEPC total and subscale scores, controlling for mother’s educational background.

**Table 4:** Pearson’s correlation coefficients between AQ and MEPC subscale scores (D–H in the CCC-2), controlling for mother’s educational background.

AQ	Total MEPC (D–H in the CCC-2)	D. Coherence	E. Inappropriate Initiation	F. Stereotyped Language	G. Use of context	H. Nonverbal Communication
Social skills	–.07 [–.18, .13]	–.07 [–.18, .13]	–.10 [–.20, .13]	–.08 [–.19, .11]	–.03 [–.15, .17]	–.05 [–.17, .14]
Attention switching	–.15 [–.24, .04]	–.10 [–.20, .09]	–.15 [–.24, .04]	–.16 [–.25, .03]	–.11 [–.21, .08]	–.17 [–.26, .02]
Local details	–.09 [–.19, .11]	–.08 [–.19, .12]	–.08 [–.19, .12]	–.07 [–.18, .13]	–.07 [–.18, .12]	–.10 [–.20, .10]
Communication	–.28** [–.34, –10]	–.23* [–.30, –03]	–.26** [–.33, –07]	–.32** [–.37, –14]	–.26* [–.32, –07]	–.24* [–.31, –05]
Imagination	–.33*** [–.38, –15]	–.28** [–.34, –09]	–.28** [–.34, –10]	–.32** [–.37, –13]	–.33*** [–.37, –14]	–.31** [–.36, –13]

Numbers in brackets are 95% confidence intervals.

*p< 0.05,

**p< 0.01,

***p< 0.001

Consistently significant negative correlations were found for the communication and imagination subscales of the AQ and MEPC D, E, F, G, and H subscale scores. According to the guidelines of Cohen [26], the correlation coefficients between most of the imagination subscales of the AQ and MEPC total scores were moderate. On the other hand, those between many of the communication subscales of the AQ and MEPC total scores were small; the correlation coefficient between the imagination subscale of the AQ and the MEPC total score was small to moderate. Next, the subscales of the AQ and MEPC other than the target subscales were used as control variables and the partial correlation coefficients were calculated. As a result, the partial correlation coefficients ranged from –0.18 to 0.11, neither of which were significant.

### Examination of excluded similar items

To consider the possibility that significant negative correlations in the current data occurred because of the similarity of AQ and CCC-2 items, we examined correlations after excluding three sets of similar items. However, significant negative correlations remained between the 47 AQ and 22 MEPC items (*r* = –0.25, *p* = 0.01). In addition, significant negative correlations were observed between the communication subscale of the AQ and the 22 MEPC subscales (*r* = –0.28, *p* < 0.01). There was no item similar to the MEPC on the imagination subscale of the AQ.

### Examination excluding outliers

Next, correlation coefficients were calculated, except for in two mothers whose AQ scores exceeded the cutoff point. Even after excluding the outliers, a significant correlation remained between the AQ total and MEPC total scores (*n* = 98, *r* = –0.28, *p* < 0.01).

### Comparing the correlation between maternal AQ and MEPC scores by assumed child’s gender

A marginally significant negative correlation was observed between maternal AQ total and MEPC total scores in both genders (boys: *r* = –0.06, *p* = 0.09; girls: *r* = –0.25, *p* = 0.08). Comparing the correlation coefficients between the AQ total and MEPC total scores in cases where the assumed children were boys and girls, no significant difference was found (*z* = 0.967, *p* = 0.34). Cohen’s q, which represents the effect size of the difference between the correlation coefficients, was 0.2, which is considered a small effect.

### Correlation between maternal AQ and MEPC scores by assumed child’s age

Differences according to the age of the child presumed by mothers when responding to the MEPC were observed. Although a marginally significant negative correlation (*r* = –0.26, *p* = 0.06) was found between maternal AQ and MEPC scores in assumed children in lower grades (7–9 years of age), a significant negative correlation (*r* = –0.29, *p* < 0.05) was observed in assumed children in higher grades (10–12 years of age). Comparing the correlation between the AQ total and MEPC total scores when the age of assumed children was 7–9 vs. 10–12 years, no significant difference was found (*z* = 0.12, *p* = 0.91). The effect size index q was 0.02, which was evaluated as having no effect.

## Discussion

In general, the more mothers with higher AQ scores, the more the pragmatic impairments of the assumed children are evaluated as being no problem. No significant effect was found on the correlation between AQ total and MEPC total scores depending on the mother’s educational level or gender and age of assumed children. The first way to explain this result is the similarity hypothesis. The results of the present study suggest that higher maternal AQ scores among mothers predict a greater degree of permissiveness to pragmatic impairments; that is, the BAP implied lower MEPC scores. This viewpoint is in agreement with previous research by Komeda et al. [13], who reported that higher AQ scores in ASD and TD individuals were significantly correlated with greater activation in the vmPFC while judging characters with ASD traits, meaning that individuals with a high level of ASD traits tend to empathize with similar others. Komeda et al. [13] concluded that individuals with ASD do not lack empathy toward others who are similar to themselves, just as TD individuals respond selectively to others who are similar compared with those who are dissimilar. We therefore suggest that the AQ and pragmatic impairments have relatively close underlying structures because a correlation was observed between AQ and MEPC scores, even after excluding similar items.

However, there are many possibilities other than empathy as a factor that regards assumed children’s pragmatic impairments as not being a problem. Mothers with ASD traits are sometimes unaware of their autism traits and reckon that these behaviors are “not a problem”. Alternatively, self-esteem and coping resources may allow some mothers to feel more than capable to manage a behavior and consider it as not being a problem. Some ideologies about breeding may normalize a lot of child behaviors, so many amount alternative explanation could be explored.

Moreover, the AQ is not the only factor affecting MEPC scores. We also investigated the influence of assumed age, gender, mother’s occupation, and other factors on MEPC scores. A significant difference was found in the degree of the evaluation between children assumed to be 7–9 vs. those assumed to be 10–12 years of age when the mother evaluated pragmatic impairments. We interpreted this as meaning that mothers evaluated pragmatic impairments as problematic when the assumed children were older because there are more expectations in terms of a child’s communicative competence. Further research with a larger sample is required to clarify how mothers evaluate pragmatic impairments in assumed children according to age. No significant differences were observed between the gender of assumed children and MEPC scores, but a small effect was seen on the correlation coefficient between the AQ total and MEPC total scores when the assumed children were boys or girls. Further research with a larger sample is required to clarify how mothers evaluate pragmatic impairments in assumed children according to gender. Other factors such as the birth order of children, the presence or absence and number of siblings, the maternal socioeconomic status level, and the mother’s own birth situation, also warrant consideration.

The result that higher maternal AQ scores predict lower MEPC scores is both positive and negative. The positive aspect is that there is a possibility that the higher the mother’s AQ, the less she is expected to be irritated by or to scold the child for his/her pragmatic impairments. The negative aspect is that it is likely that special support would not reach the child with pragmatic impairments in need because parents who are more tolerant of pragmatic impairments in their children would consider them to be less of a problem, and therefore less perceivable.

The possibility remains that it was difficult for mothers with higher AQ scores to complete the questionnaires with the presumption that their child showed pragmatic impairments. In this case, the results did not reflect the permissiveness of the mothers, but rather the incapacity to keep in mind the possible pragmatic impairments of the child and the implications of these impairments for everyday life. Since few prior studies have evaluated assumed impairments, these possibilities should be considered in future investigations.

The present study had four major limitations. First, the presence of ASD in mothers was unknown. Second, the presence of ASD or other neurodevelopmental disorders in their children was unknown. Third, while we focused on the evaluation of children’s assumed pragmatic impairments at the time of the questionnaire, there was no way to know if the mother had evaluated their actual pragmatic impairments. Fourth, the A, B, C, I, and J subscales of the CCC-2 should also be examined in relation to maternal AQ scores. Examining the correlation between maternal AQ scores and each composite of the CCC-2—pragmatics (D–H), structural language (A–D), and autistic type behaviors (I and J)—would help to clarify which composites are correlated with maternal AQ. Future tasks include considering study subjects with fathers and examining relationships in relation to friendships and the workplace using the Communication Checklist–Adult version [30].

## Conclusions

The findings of the present study suggest that among mothers with normal elementary school children, those with higher AQ scores tend to estimate the pragmatic impairments of assumed children as not being problematic, and are therefore more permissive of children with pragmatic impairments. Another factor that affected MEPC scores was the age of assumed children. In the case of higher grades (10–12 years), the pragmatic impairments of assumed children were estimated as being problematic by the mothers. Therefore, further investigations with an increased number of samples regarding whether the age and gender of assumed children affect the correlation between AQ and MEPC scores are needed. There could many reasons why mothers with higher AQ scores are more permissive of an assumed child’s pragmatic impairments, but the findings of the present study, although incomplete, are worth considering in the daily clinical setting.

## Supporting information

S1 Fig Correlation between maternal AQ and MEPC scores (*r* = –0.26, *p* < 0.01). (TIF)

S1 File Raw data. (XLSX)

S2 File Question correspondence table of raw data. (PDF)

S3 File Partial correlation coefficients and p values between subscales. (XLSX)
