# Supplementary material for: Association between maternal Autism Spectrum Quotient scores and the tendency to see pragmatic impairments as a problem
Source: PLoS One. 2018 Dec 19;13(12):e0209412. doi: 10.1371/journal.pone.0209412 (PMC6300330; doi:10.1371/journal.pone.0209412)
Supplement: S2 File — (PDF) [file pone.0209412.s003.pdf]

	SC1. あなたの性別をお知らせください。（1つ選択）	SA
1	男性	
2	女性	

	SC2. あなたの年齢をお知らせください。（数値記入）	NA

	SC3. 現在お住まいの都道府県をお知らせください。（1つ選択）	SA
1	北海道	
2	青森県	
3	岩手県	
4	宮城県	
5	秋田県	
6	山形県	
7	福島県	
8	茨城県	
9	栃木県	
10	群馬県	
11	埼玉県	
12	千葉県	
13	東京都	
14	神奈川県	
15	新潟県	
16	富山県	
17	石川県	
18	福井県	
19	山梨県	
20	長野県	
21	岐阜県	
22	静岡県	
23	愛知県	
24	三重県	
25	滋賀県	
26	京都府	
27	大阪府	
28	兵庫県	
29	奈良県	
30	和歌山県	
31	鳥取県	
32	島根県	
33	岡山県	
34	広島県	
35	山口県	
36	徳島県	
37	香川県	
38	愛媛県	
39	高知県	
40	福岡県	
41	佐賀県	
42	長崎県	
43	熊本県	
44	大分県	
45	宮崎県	
46	鹿児島県	
47	沖縄県	

	SC3-2. あなたの婚姻状況をお知らせください。（1つ選択）	SA
1	未婚	
2	既婚	
3	離死別	

	SC4. あなたの職業をお知らせください。（1つ選択）	SA
1	経営者・役員	
2	会社員（管理職）	
3	会社員（正社員）	
4	会社員（派遣・契約社員）	
5	公務員・教職員	
6	専門職、士師業・フリーランス	

7	自営業	
8	パートタイム･アルバイト	
9	専業主婦（主夫）	
10	学生	
11	その他	

	SC5. あなたの最終学歴をお知らせください。（1つ選択）	SA
1	中学校	
2	高校	
3	専門学校	
4	短期大学	
5	大学	
6	大学院	
7	その他	

	SC6. あなたにはお子様が何人いらっしゃいますか。（1つ選択）	SA
1	子供はいない	
2	1人	
3	2人	
4	3人	
5	4人	
6	5人以上	

	SC7. あなたのお子様の性別と年齢と学齢をお知らせください。※第一子から順にお答えください。	
	SC7-1-1. 第一子――性別	SA
1	男の子	
2	女の子	

	SC7-2-1. 第一子――年齢	NA

	SC7-3-1. 第一子――学齢	SA
1	未就学児	
2	幼稚園生/保育園生	
3	小学1年生	
4	小学2年生	
5	小学3年生	
6	小学4年生	
7	小学5年生	
8	小学6年生	
9	中学1年生	
10	中学2年生	
11	中学3年生	
12	高校1年生	
13	高校2年生	
14	高校3年生	
15	短大生／専門学校生	
16	大学生／大学院生	
17	社会人	
18	その他	

	SC7-1-2. 第二子――性別	SA
1	男の子	
2	女の子	

	SC7-2-2. 第二子――年齢	NA

	SC7-3-2. 第二子――学齢	SA
1	未就学児	
2	幼稚園生/保育園生	
3	小学1年生	
4	小学2年生	
5	小学3年生	
6	小学4年生	
7	小学5年生	
8	小学6年生	
9	中学1年生	

10	中学2年生
11	中学3年生
12	高校1年生
13	高校2年生
14	高校3年生
15	短大生／専門学校生
16	大学生／大学院生
17	社会人
18	その他

	SC7-1-3. 第三子――性別	SA
1	男の子	
2	女の子	

	SC7-2-3. 第三子――年齢	NA

	SC7-3-3. 第三子――学齢	SA
1	未就学児	
2	幼稚園生/保育園生	
3	小学1年生	
4	小学2年生	
5	小学3年生	
6	小学4年生	
7	小学5年生	
8	小学6年生	
9	中学1年生	
10	中学2年生	
11	中学3年生	
12	高校1年生	
13	高校2年生	
14	高校3年生	
15	短大生／専門学校生	
16	大学生／大学院生	
17	社会人	
18	その他	

	SC7-1-4. 第四子――性別	SA
1	男の子	
2	女の子	

	SC7-2-4. 第四子――年齢	NA

	SC7-3-4. 第四子――学齢	SA
1	未就学児	
2	幼稚園生/保育園生	
3	小学1年生	
4	小学2年生	
5	小学3年生	
6	小学4年生	
7	小学5年生	
8	小学6年生	
9	中学1年生	
10	中学2年生	
11	中学3年生	
12	高校1年生	
13	高校2年生	
14	高校3年生	
15	短大生／専門学校生	
16	大学生／大学院生	
17	社会人	
18	その他	

	SC7-1-5. 第五子――性別	SA
1	男の子	
2	女の子	

	SC7-2-5. 第五子――年齢	NA

	SC7-3-5. 第五子――学齢	SA
1	未就学児	
2	幼稚園生/保育園生	
3	小学1年生	
4	小学2年生	
5	小学3年生	
6	小学4年生	
7	小学5年生	
8	小学6年生	
9	中学1年生	
10	中学2年生	
11	中学3年生	
12	高校1年生	
13	高校2年生	
14	高校3年生	
15	短大生／専門学校生	
16	大学生／大学院生	
17	社会人	
18	その他	

	Q1. 以下のそれぞれの項目について、あなたにあてはまるものを1つずつお選びください。（それぞれ1つずつ選択）	
	Q1-1. 何かをするときには、一人でするよりも他の人といっしょにすることを好む。	SA
1	あてはまる	
2	どちらかといえば、あてはまる	
3	どちらかといえば、あてはまらない	
4	あてはまらない（ちがう）	

	Q1-2. 同じことを（同じやりかたで）、何度もくりかえすことが好きだ。	SA
1	あてはまる	
2	どちらかといえば、あてはまる	
3	どちらかといえば、あてはまらない	
4	あてはまらない（ちがう）	

	Q1-3. 何かを想像しようとすれば、その映像（イメージ）を簡単に思い浮かべること	SA
1	あてはまる	
2	どちらかといえば、あてはまる	
3	どちらかといえば、あてはまらない	
4	あてはまらない（ちがう）	

	Q1-4. 一つのことに没頭して、ほかのことがぜんぜん目に入らなくなる（気がつかなくなる）ことがよくある。	SA
1	あてはまる	
2	どちらかといえば、あてはまる	
3	どちらかといえば、あてはまらない	
4	あてはまらない（ちがう）	

	Q1-5. 他の人は気がつかないような、小さな物音に気がつくことがしばしばある。	SA
1	あてはまる	
2	どちらかといえば、あてはまる	
3	どちらかといえば、あてはまらない	
4	あてはまらない（ちがう）	

	Q1-6. 車のナンバーや時刻表の数字などといった一連の数字などの情報に注意が向くことがよくある。	SA
1	あてはまる	
2	どちらかといえば、あてはまる	
3	どちらかといえば、あてはまらない	
4	あてはまらない（ちがう）	

	Q1-7. 自分ではていねいに話したつもりでも、話し方が失礼だと周囲の人から言われることがよくある。	SA
1	あてはまる	
2	どちらかといえば、あてはまる	
3	どちらかといえば、あてはまらない	
4	あてはまらない（ちがう）	

	Q1-8. 小説(物語)などを読んでいるとき、登場人物の外見がどんな人かについて簡単に想像することができる。	SA
1	あてはまる	
2	どちらかといえば、あてはまる	
3	どちらかといえば、あてはまらない	
4	あてはまらない(ちがう)	

	Q1-9. 日付・曜日などについてのこだわりがある。	SA
1	あてはまる	
2	どちらかといえば、あてはまる	
3	どちらかといえば、あてはまらない	
4	あてはまらない(ちがう)	

	Q1-10. パーティーや会合などで、いろいろな(複数の)人の会話についていくことが簡単にできる。	SA
1	あてはまる	
2	どちらかといえば、あてはまる	
3	どちらかといえば、あてはまらない	
4	あてはまらない(ちがう)	

	Q1-11. たくさんの人がいる状況や場面でも緊張することはない。	SA
1	あてはまる	
2	どちらかといえば、あてはまる	
3	どちらかといえば、あてはまらない	
4	あてはまらない(ちがう)	

	Q1-12. ほかの人は気がつかないような細かいことに気づくことが多い。	SA
1	あてはまる	
2	どちらかといえば、あてはまる	
3	どちらかといえば、あてはまらない	
4	あてはまらない(ちがう)	

	Q1-13. パーティーなどよりも、図書館に行く方が好きだ。	SA
1	あてはまる	
2	どちらかといえば、あてはまる	
3	どちらかといえば、あてはまらない	
4	あてはまらない(ちがう)	

	Q1-14. 新しい話(ストーリー)を、すぐにつくることができる。	SA
1	あてはまる	
2	どちらかといえば、あてはまる	
3	どちらかといえば、あてはまらない	
4	あてはまらない(ちがう)	

	Q1-15. モノよりも人間の方に魅力を感じる。	SA
1	あてはまる	
2	どちらかといえば、あてはまる	
3	どちらかといえば、あてはまらない	
4	あてはまらない(ちがう)	

	Q1-16. それをすることができないと、ひどく取り乱したり興奮してしまうくらい強い興味や関心を持っていること(もの)がある。	SA
1	あてはまる	
2	どちらかといえば、あてはまる	
3	どちらかといえば、あてはまらない	
4	あてはまらない(ちがう)	

	Q1-17. 人とちょっとした会話(おしゃべり)を楽しむことができる。	SA
1	あてはまる	
2	どちらかといえば、あてはまる	
3	どちらかといえば、あてはまらない	
4	あてはまらない(ちがう)	

	Q1-18. 自分が話をしているときには、なかなか他の人に横から口をはさませな	SA
1	あてはまる	
2	どちらかといえば、あてはまる	

3	どちらかといえば、あてはまらない	
4	あてはまらない（ちがう）	

	Q1-19. 数字や番号についてのこだわりがある。	SA
1	あてはまる	
2	どちらかといえば、あてはまる	
3	どちらかといえば、あてはまらない	
4	あてはまらない（ちがう）	

	Q1-20. 小説などを読んだり、テレビドラマなどを観ているとき、登場人物の意図や考えなどをよく理解できないことがある。	SA
1	あてはまる	
2	どちらかといえば、あてはまる	
3	どちらかといえば、あてはまらない	
4	あてはまらない（ちがう）	

	Q1-21. 小説などのようなフィクションの本を読むことは、あまり好きではない。	SA
1	あてはまる	
2	どちらかといえば、あてはまる	
3	どちらかといえば、あてはまらない	
4	あてはまらない（ちがう）	

	Q1-22. 新しい友人を作ることは、苦手である。	SA
1	あてはまる	
2	どちらかといえば、あてはまる	
3	どちらかといえば、あてはまらない	
4	あてはまらない（ちがう）	

	Q1-23. いつでも、ものごとの中に何らかのパターン（型や法則など）のようなものがあることに気づく。	SA
1	あてはまる	
2	どちらかといえば、あてはまる	
3	どちらかといえば、あてはまらない	
4	あてはまらない（ちがう）	

	Q1-24. 博物館に行くよりも，劇場や映画館に行く方が好きだ。	SA
1	あてはまる	
2	どちらかといえば、あてはまる	
3	どちらかといえば、あてはまらない	
4	あてはまらない（ちがう）	

	Q1-25. 自分のいつもの日課(行動の順序など）がじゃまされても、取り乱すようなことはない。	SA
1	あてはまる	
2	どちらかといえば、あてはまる	
3	どちらかといえば、あてはまらない	
4	あてはまらない（ちがう）	

	Q1-26. 会話をどのように続けたらいいのか、わからなくなってしまうことがよくあ	SA
1	あてはまる	
2	どちらかといえば、あてはまる	
3	どちらかといえば、あてはまらない	
4	あてはまらない（ちがう）	

	Q1-27. 誰かと話をしているときに、相手の話の‘言外の意味‘を容易に理解することができる。	SA
1	あてはまる	
2	どちらかといえば、あてはまる	
3	どちらかといえば、あてはまらない	
4	あてはまらない（ちがう）	

	Q1-28. ものごとの細かいところよりも、全体像に注意が向くことが多い。	SA
1	あてはまる	
2	どちらかといえば、あてはまる	
3	どちらかといえば、あてはまらない	
4	あてはまらない（ちがう）	

	Q1-29. 電話番号をおぼえるのは苦手である。	SA
1	あてはまる	
2	どちらかといえば、あてはまる	
3	どちらかといえば、あてはまらない	
4	あてはまらない（ちがう）	

	Q1-30. 状況（部屋の様子やものの置き場所など）や人間の外見（服装や髪型）などが、いつもとちょっと違っているくらいでは、すぐには気がつかないことが多い。	SA
1	あてはまる	
2	どちらかといえば、あてはまる	
3	どちらかといえば、あてはまらない	
4	あてはまらない（ちがう）	

	Q1-31. 自分の話を聞いている相手が退屈しているときには、どのように話をすればいいのかわかっている。	SA
1	あてはまる	
2	どちらかといえば、あてはまる	
3	どちらかといえば、あてはまらない	
4	あてはまらない（ちがう）	

	Q1-32. 同時に2つ以上のことをするのは、容易である。	SA
1	あてはまる	
2	どちらかといえば、あてはまる	
3	どちらかといえば、あてはまらない	
4	あてはまらない（ちがう）	

	Q1-33. 電話で話をしているとき、自分が話をするタイミングがわからないことがあ	SA
1	あてはまる	
2	どちらかといえば、あてはまる	
3	どちらかといえば、あてはまらない	
4	あてはまらない（ちがう）	

	Q1-34. 自分から進んで(自発的に)何かをすることは楽しい。	SA
1	あてはまる	
2	どちらかといえば、あてはまる	
3	どちらかといえば、あてはまらない	
4	あてはまらない（ちがう）	

	Q1-35. 冗談がわからないことがよくある。	SA
1	あてはまる	
2	どちらかといえば、あてはまる	
3	どちらかといえば、あてはまらない	
4	あてはまらない（ちがう）	

	Q1-36. 相手の顔を見れば、その人が考えていることや感じていることがわかる。	SA
1	あてはまる	
2	どちらかといえば、あてはまる	
3	どちらかといえば、あてはまらない	
4	あてはまらない（ちがう）	

	Q1-37. 何かをしているときに、じゃまが入っても、すぐにそれまでやっていたことに戻ることができる。	SA
1	あてはまる	
2	どちらかといえば、あてはまる	
3	どちらかといえば、あてはまらない	
4	あてはまらない（ちがう）	

	Q1-38. 雑談や、ちょっとしたおしゃべりを人とすることが得意だ。	SA
1	あてはまる	
2	どちらかといえば、あてはまる	
3	どちらかといえば、あてはまらない	
4	あてはまらない（ちがう）	

	Q1-39. 同じことを何度も繰り返していると、周囲の人からよく言われる。	SA
1	あてはまる	
2	どちらかといえば、あてはまる	
3	どちらかといえば、あてはまらない	

4	あてはまらない（ちがう）	

	Q1-40. 子どものころ、友達といっしょに「〇〇ごっこ」（ごっこ遊び）をよくして遊んで	SA
1	あてはまる	
2	どちらかといえば、あてはまる	
3	どちらかといえば、あてはまらない	
4	あてはまらない（ちがう）	

	Q1-41. 特定の種類(カテゴリー)のもの（たとえば、車、鳥、昆虫など）についての情報を集めることが好きだ。	SA
1	あてはまる	
2	どちらかといえば、あてはまる	
3	どちらかといえば、あてはまらない	
4	あてはまらない（ちがう）	

	Q1-42. 他の人がどのように感じているかを想像をすることは苦手だ。	SA
1	あてはまる	
2	どちらかといえば、あてはまる	
3	どちらかといえば、あてはまらない	
4	あてはまらない（ちがう）	

	Q1-43. 自分がすることは、どんなことでも注意深く計画するのが好きだ。	SA
1	あてはまる	
2	どちらかといえば、あてはまる	
3	どちらかといえば、あてはまらない	
4	あてはまらない（ちがう）	

	Q1-44. 社交的な（人と親しく交わる）場面は楽しい。	SA
1	あてはまる	
2	どちらかといえば、あてはまる	
3	どちらかといえば、あてはまらない	
4	あてはまらない（ちがう）	

	Q1-45. 他の人の考え（意図など）を理解することは苦手だ。	SA
1	あてはまる	
2	どちらかといえば、あてはまる	
3	どちらかといえば、あてはまらない	
4	あてはまらない（ちがう）	

	Q1-46. 新しい場面（状況）では不安を感じやすい。	SA
1	あてはまる	
2	どちらかといえば、あてはまる	
3	どちらかといえば、あてはまらない	
4	あてはまらない（ちがう）	

	Q1-47. 初対面の人と会うことは楽しい。	SA
1	あてはまる	
2	どちらかといえば、あてはまる	
3	どちらかといえば、あてはまらない	
4	あてはまらない（ちがう）	

	Q1-48. 社交的である。	SA
1	あてはまる	
2	どちらかといえば、あてはまる	
3	どちらかといえば、あてはまらない	
4	あてはまらない（ちがう）	

	Q1-49. 家族や友人などの誕生日をおぼえるのは苦手だ。	SA
1	あてはまる	
2	どちらかといえば、あてはまる	
3	どちらかといえば、あてはまらない	
4	あてはまらない（ちがう）	

	Q1-50. 子どもと「ごっこ遊び」をして遊ぶのがとても得意だ。	SA
1	あてはまる	
2	どちらかといえば、あてはまる	
3	どちらかといえば、あてはまらない	

4 あてはまらない（ちがう）

	Q2. もし、あなたの小学生のお子さんが説明にあるような行動をした場合、保護者であるあなたはどの程度「問題である」と感じるかについて、それぞれの項目について、あてはまるものを1つずつお選びください。（それぞれ1つずつ選択）
	Q2-1. 誰も興味をもたないようなことを繰り返ししゃべる。 SA
1	問題ではない
2	あまり問題ではない
3	どちらでもない
4	やや問題である
5	問題である

	Q2-2. ほとんどの子どもが、見てそれとわかる表情（例えば怒ったり、こわがったり、嬉しがったりなど）を見せるような場面でも、無表情でいる。
1	問題ではない
2	あまり問題ではない
3	どちらでもない
4	やや問題である
5	問題である

SA

	Q2-3. 「その人」や「それ」といった表現を、対象を明らかにしないで話す。
1	問題ではない
2	あまり問題ではない
3	どちらでもない
4	やや問題である
5	問題である

SA

	Q2-4. 意味をよくわからないまま、大人が使った表現をまねているかのように言葉を使うことがある。
1	問題ではない
2	あまり問題ではない
3	どちらでもない
4	やや問題である
5	問題である

SA

	Q2-5. 相手を見ないで話す。
1	問題ではない
2	あまり問題ではない
3	どちらでもない
4	やや問題である
5	問題である

SA

	Q2-6. どたばたのお笑いなどの非言語的なユーモアを楽しむことはできるが、言葉による冗談やだじゃれの意味がわかっていない。
1	問題ではない
2	あまり問題ではない
3	どちらでもない
4	やや問題である
5	問題である

SA

	Q2-7. お気に入りのフレーズや文、長い文章を、ふさわしくない場面で使う。
1	問題ではない
2	あまり問題ではない
3	どちらでもない
4	やや問題である
5	問題である

SA

	Q2-8. 単語がいつもと違う意味で使われると混乱する。
1	問題ではない
2	あまり問題ではない
3	どちらでもない
4	やや問題である
5	問題である

SA

	Q2-9. 話をするとき、相手に近寄り過ぎる。
1	問題ではない
2	あまり問題ではない

3 どちらでもない
4 やや問題である
5 問題である

SA

Q2-10. 周りから促されなくても知らない人と会話を始めてしまうなど、他人にあまりにも気安く話しかける。
1 問題ではない
2 あまり問題ではない
3 どちらでもない
4 やや問題である
5 問題である

SA

Q2-11. 過剰なほどに正確に発音する。そのため、身近な人というより、アナウンサーをまねしているような不自然な口調になる。
1 問題ではない
2 あまり問題ではない
3 どちらでもない
4 やや問題である
5 問題である

SA

Q2-12. 本当の話をしているのか、想像の話をしているのか、わからないような話し方をする。
1 問題ではない
2 あまり問題ではない
3 どちらでもない
4 やや問題である
5 問題である

SA

Q2-13. コミュニケーションがうまくできる場面とできない場面がある。
1 問題ではない
2 あまり問題ではない
3 どちらでもない
4 やや問題である
5 問題である

SA

Q2-14. 他の人が言ったばかりのことを繰り返す。
1 問題ではない
2 あまり問題ではない
3 どちらでもない
4 やや問題である
5 問題である

SA

Q2-15. 他の人から話しかけられても無視する。
1 問題ではない
2 あまり問題ではない
3 どちらでもない
4 やや問題である
5 問題である

SA

Q2-16. 相手の言ったことのうち、１つか２つの単語しか聞かず、誤解することがあ
1 問題ではない
2 あまり問題ではない
3 どちらでもない
4 やや問題である
5 問題である

SA

Q2-17. 話し始めると、止めるのがむずかしい。
1 問題ではない
2 あまり問題ではない
3 どちらでもない
4 やや問題である
5 問題である

SA

Q2-18. 相手が既に知っていることを話す。
1 問題ではない
2 あまり問題ではない
3 どちらでもない

4 やや問題である
5 問題である

SA

Q2-19. 相手が気分を害したり、怒っているのに気がつかない。

1 問題ではない
2 あまり問題ではない
3 どちらでもない
4 やや問題である
5 問題である

SA

Q2-20. 物語の内容や、最近の出来事について話すとき、出来事を筋道立てて話

1 問題ではない
2 あまり問題ではない
3 どちらでもない
4 やや問題である
5 問題である

SA

Q2-21. 慣用句などを文字通りに解釈してしまい、その結果、時として、意図せずおかしな言動をする。

1 問題ではない
2 あまり問題ではない
3 どちらでもない
4 やや問題である
5 問題である

SA

Q2-22. 時や場所などを必要以上に正確に表現する。

1 問題ではない
2 あまり問題ではない
3 どちらでもない
4 やや問題である
5 問題である

SA

Q2-23. 答えを知っているのに、同じ質問を繰り返す。

1 問題ではない
2 あまり問題ではない
3 どちらでもない
4 やや問題である
5 問題である

SA

Q2-24. 話の内容を初めて聞く相手に対しても、特に説明をせずに話す。

1 問題ではない
2 あまり問題ではない
3 どちらでもない
4 やや問題である
5 問題である

SA

Q2-25. 言いたいことがわかりにくい(使っている言葉は明瞭であるにもかかわら

1 問題ではない
2 あまり問題ではない
3 どちらでもない
4 やや問題である
5 問題である
